# A novel method of rapid detection for heavy metal copper ion via a specific copper chelator bathocuproinedisulfonic acid disodium salt

**DOI:** 10.1038/s41598-023-37838-y

**Published:** 2023-07-04

**Authors:** Yali Wang, Tinglin Ma, Joseph Brake, Zhaoyue Sun, Jiayu Huang, Jing Li, Xiaobin Wu

**Affiliations:** 1 Department of Chemistry and Chemical Engineering, Yulin University, Yulin, 719000 Shaanxi China; 2 Development Center of Plant Germplasm Resources, College of Life Sciences, Shanghai Normal University, Shanghai, 200234 China; 3 Department of Biochemistry and Redox Biology Center, University of Nebraska-Lincoln, Lincoln, NE 68588-0664 USA

**Keywords:** Biological techniques, Biotechnology

## Abstract

The extensive usage and production of copper may lead to toxic effects in organisms due to its accumulation in the environment. Traditional methods for copper detection are time consuming and infeasible for field usage. It is necessary to discover a real-time, rapid and economical method for detecting copper to ensure human health and environmental safety. Here we developed a colorimetric paper strip method and optimized spectrum method for rapid detection of copper ion based on the specific copper chelator bathocuproinedisulfonic acid disodium salt (BCS). Both biological assays and chemical methods verified the specificity of BCS for copper. The optimized reaction conditions were 50 mM Tris–HCl pH 7.4, 200 µM BCS, 1 mM ascorbate and less than 50 µM copper. The detection limit of the copper paper strip test was 0.5 mg/L by direct visual observation and the detection time was less than 1 min. The detection results of grape, peach, apple, spinach and cabbage by the optimized spectrum method were 0.91 μg/g, 0.87 μg/g, 0.19 μg/g, 1.37 μg/g and 0.39 μg/g, respectively. The paper strip assays showed that the copper contents of grape, peach, apple, spinach and cabbage were 0.8 mg/L, 0.9 mg/L, 0.2 mg/L, 1.3 mg/L and 0.5 mg/L, respectively. These results correlated well with those determined by inductively coupled plasma-mass spectrometry (ICP-MS). The visual detection limit of the paper strip based on Cu-BCS-AgNPs was 0.06 mg/L. Our study demonstrates the potential for on-site, rapid and cost-effective copper monitoring of foods and the environment.

## Introduction

Copper is an essential trace element from bacteria to humans but excess copper can be toxic^1,2^. Copper is widely used in industrial and agricultural fields, such as in alloys, ceramics, pesticides and electronics leading to detrimental effects in organisms due to its accumulation^3,4^. Therefore, the detection of copper contents in the environment and food is particularly important to ensure environmental safety and human health.

There are some traditional methods for effective detection of copper ions including inductively coupled plasma mass spectroscopy (ICP-MS)^5^, atomic absorption spectroscopy (AAS)^6^, X-ray fluorescence spectrometry (XRF)^7^, neutron activation analysis (NAA) and inductively coupled plasma-optical emission spectrometry (ICP-OES)^8^. Although these methods have the advantages of accuracy and sensitivity in detecting copper ion, they are costly, time consuming, complex and lack easy portability^9^. As a result, rapid, convenient detection methods are needed to monitor copper concentrations.

Over the last decade, paper-based analytical devices (PADs) have attracted increasing attention owing to their easy portability, on-site detection, simple operation, low cost and multifunctional analysis^10–12^. Many studies have shown that paper analytical test strips could successfully detect heavy metal ions in food safety, health and environmental testing^10,13^. Using a Cd − EDTA − BSA conjugate labeled with AuNPs as signal producer tool, Marzo et al. developed a highly sensitive lateral flow device with integrated sample treatment for Cd^2+^ detection in drinking and tap water samples^14^. Rattanarat et al. established three-dimensional μPADs using colorimetric and electrochemical methods to effectively detect heavy metal ions such as nickel, iron, copper, lead and chromium^15^. Furthermore, a paper-based analytical device based on colorimetric paper assays provided a portable, low cost, and lightweight method to detect Fe^2+^ in tap water^16^. Recently, Muhammad et al. fabricated a single colorimetric paper strip integrated with a smartphone providing an all-in-one device with on-site detection, leading to cost-effective and rapid assays for the detection of Zn, Cr, Cu, Pb and Mn in wastewater^11^. However, in order to remove the interference of other heavy metal ions, sodium cyanide, a highly toxic substance, was added to the paper strip, which is likely to cause secondary environmental pollution and harm to testing personnel.

In general, the detection of heavy metal ions by paper analytical test strips mainly use combinations of heavy metal ions and chelators to produce color reactions. However, the current paper analytical test strips need further improvement due to the defects of low specificity, toxicity and weak binding ability of chelators. Therefore, screening of heavy metal ion chelators with high specificity, low toxicity, strong binding ability and ability to produce a color reaction is crucial to preparing paper test strips of practical use in the detection of trace heavy metal ions.

Bathocuproinedisulfonic acid disodium salt (BCS) has been used by others as a copper chelator for measurement of Cu ions and Cu-protein complexes^17,18^. The BCS-Cu^+^ complex has an absorption peak at 490 nm with good linearity when used for spectrophotometric detection of copper^19–21^. However, there is insufficient information about the specificity of BCS for copper for its use in PAD development. In this study, we used metal-sensitive yeast knockout strains to assay for the metal specificity of BCS. Ace1p, a yeast transcription factor for alleviating copper toxicity, binds with Cu(I) to initiate the expression of genes which encode copper binding metallothioneins and Sod1p^22–25^. Accordingly, *ACE1* knock-out cells (*ace1Δ*) are extremely sensitive to excess copper. Other metal sensitive yeast strains (*pca1Δ*, *ftr1Δ*, *fet3Δ*) and wild type yeast were used for toxicity assays of other heavy metals^22,26^.

For the first time, we demonstrated recovery of copper-treated *ace1Δ* cells by BCS, while none of the other metal-sensitive strains were recovered by BCS when treated with toxic concentrations of other heavy metals.

We further characterized the BCS-Cu^+^ complex and found it developed a yellow color in solution with color intensity proportional to the copper contents. We optimized the reaction conditions with respect to buffer, pH, BCS and ascorbate to maximize the absorbance peak at 490 nm. The colorimetric reaction was also successfully quantitated using a paper strip method with under 1 min detection time. We next demonstrated the applicability of the method by accurately determining the copper content of several fruits and vegetables using our reaction system (both optimized spectrum measurement and paper strip methods) which was verified by comparison to ICP-MS results. Moreover, a Cu-BCS-silver nanoparticles (Cu-BCS-AgNPs) system was established to further improve the color change of standard paper method and decrease the detection limit. We demonstrated a novel rapid, on-site, low-cost and safe single paper strip detection method for copper.

## Materials and methods

All chemical reagents were obtained from Sigma-Aldrich (China), except special instructions.

### Yeast strains, culture media, and growth assays

A haploid control yeast S. cerevisiae strain, BY4741, and knock out mutants^27^ were purchased from Open Biosystems. *ace1Δ*, *pca1Δ*, *ftr1Δ* and *fet3Δ* and wild type strains were used to test the sensitivities of Cu^+^, Cd^2+^, Ni^2+^, Pb^2+^, Cr^3+^ and Hg^2+^, respectively. Yeast cells were cultured in synthetic complete media (SC) and 1.5% agar was supplemented into the liquid media for solid medium plates. Yeast strains were cultured at 30 °C. For yeast cell growth assays^28,29^, WT or knock out cells were grown over-night in SC media and re-inoculated (OD_600_ = 0.2) into fresh media, and grown to mid-log phase (OD_600_ = 0.8–1.0). After dilution to OD_600_ = 0.1 and 3 × serial dilutions in sterilized water, ~ 5 µL of cells were spotted on SC plates supplemented with various amounts of CuSO_4_, CdCl_2_, NiSO_4_, CrCl_3_, Hg(NO_3_)_2_ and Pb(NO_3_)_2_ with or without BCS or the non-specific metal chelator EDTA. Cells were grown at 30 °C for 2–3 days prior to photography. Each assay was repeated at least three times using three different colonies to confirm results.

### Optimization of BCS assay

The BCS assay was optimized from the previous study^19^. Since BCS only forms a complex with cuprous ions with a maximum absorbance at 490 nm, we added ascorbate to reduce all copper into cuprous from. To optimize the BCS assay condition, we compared the reaction system under varying buffer type (Tris–HCl, PBS, MES), pH (6.5, 7.4, 8.5), ascorbate (0, 0.02, 0.1, 0.25, 1 mM), BCS (0, 10, 50, 100, 200 μM) and Cu (0, 25, 50 μM). The reaction time was less than 1 min.

### Preparation of standard curve

To quantify the BCS-Cu^+^ color on paper, the optimized reaction condition (50 mM Tris–HCl pH 7.4, 200 μΜ BCS, 1 mM ascorbate) was used to make a standard curve. A series of CuSO_4_ concentrations (0, 0.2, 0.5, 1. 2. 5, 10, 20 mg/L) were added into the reaction system and dripped on paper (0.5 cm × 6 cm of standard filter paper). The reaction solutions were then measured at 490 nm by spectrophotometry.

### Sample preparation for color reaction and ICP-MS measurement

0.1 g sample (grape, peach, apple, spinach, cabbage) was collected and smashed into juice^30^. The juice samples were dissolved in 50 μL 10% nitric acid at room temperature for 20 min and subsequently the whole solution was taken into the reaction system for 490 nm measurement and paper test respectively.

For ICP-MS measurement, the juice samples were dissolved in 70% nitric acid at 70 °C for 3 h and then overnight at room temperature and subsequently diluted in 10% nitric acid. ICP-MS (Agilent Model 7500cs, Santa Clara, CA) was used to quantify metal ions. Metal ion contents were normalized to sample weight^31,32^.

### Synthesis of BCS-AgNPs

Silver nanoparticles were synthesized by reducing AgNO_3_ with NaBH_4_ as described elsewhere with minor modifications^33^. 250 μL AgNO_3_ (0.05 M) was added to 50 mL of ultrapure water and stirred well at room temperature. Then, 250 μL of 0.1 M freshly prepared NaBH_4_ solution was quickly added to the mixed solution and stirred on a magnetic stirrer in the dark for 5 min. The solution changed from colorless to bright yellow indicating the formation of a bright yellow silver colloidal solution. Next, 500 μL of 0.05 M BCS dissolved in water was added to the solution and subsequently stirred 2 h in the dark. The solution color switched from bright yellow to light yellow. Finally, the prepared BCS-AgNPs solution was stored at 4 °C in the dark.

### Characterization of BCS-AgNPs

The naked eye observation revealed a light yellow color, indicating the formation of BCS-AgNPs. This was further confirmed by conducting UV full-wavelength scanning with a 1.0 cm quartz cell using a UV spectrometer. The absorption spectrum of AgNPs exhibited a distinct peak at 390 nm. The peak shifted to 410 nm and a characteristic absorption peak of BCS-Cu was formed at 490 nm after BCS addition. The ratio of A490 nm/A410 nm served as a parameter for the quantitative detection of Cu^+^. 10 μL AgNPs solution was placed on a carbon-coated copper grid (300 mesh) for transmission electron microscopy (TEM, JEM-2100) to observe the morphology of the silver nanoparticles in different systems and a particle size distribution map was prepared. The BCS-AgNPs solution was placed in a refrigerated vacuum dryer for 48 h. The dried material was subjected to FTIR (FTIR-7600) analysis in KBr particles within the range of 500–4000 cm^-1^.

### Statistical analysis

Descriptive analyses were presented as the mean ± S.D. and statistical comparisons of control and experimental groups were performed using Student’s t-test. p < 0.05 was considered to be significant.

### Ethics guideline statement

The collection of plant material, comply with relevant institutional, national, and international guidelines and legislation.

## Results

### A novel biological assay to test BCS binding specificity

**Figure 1:**
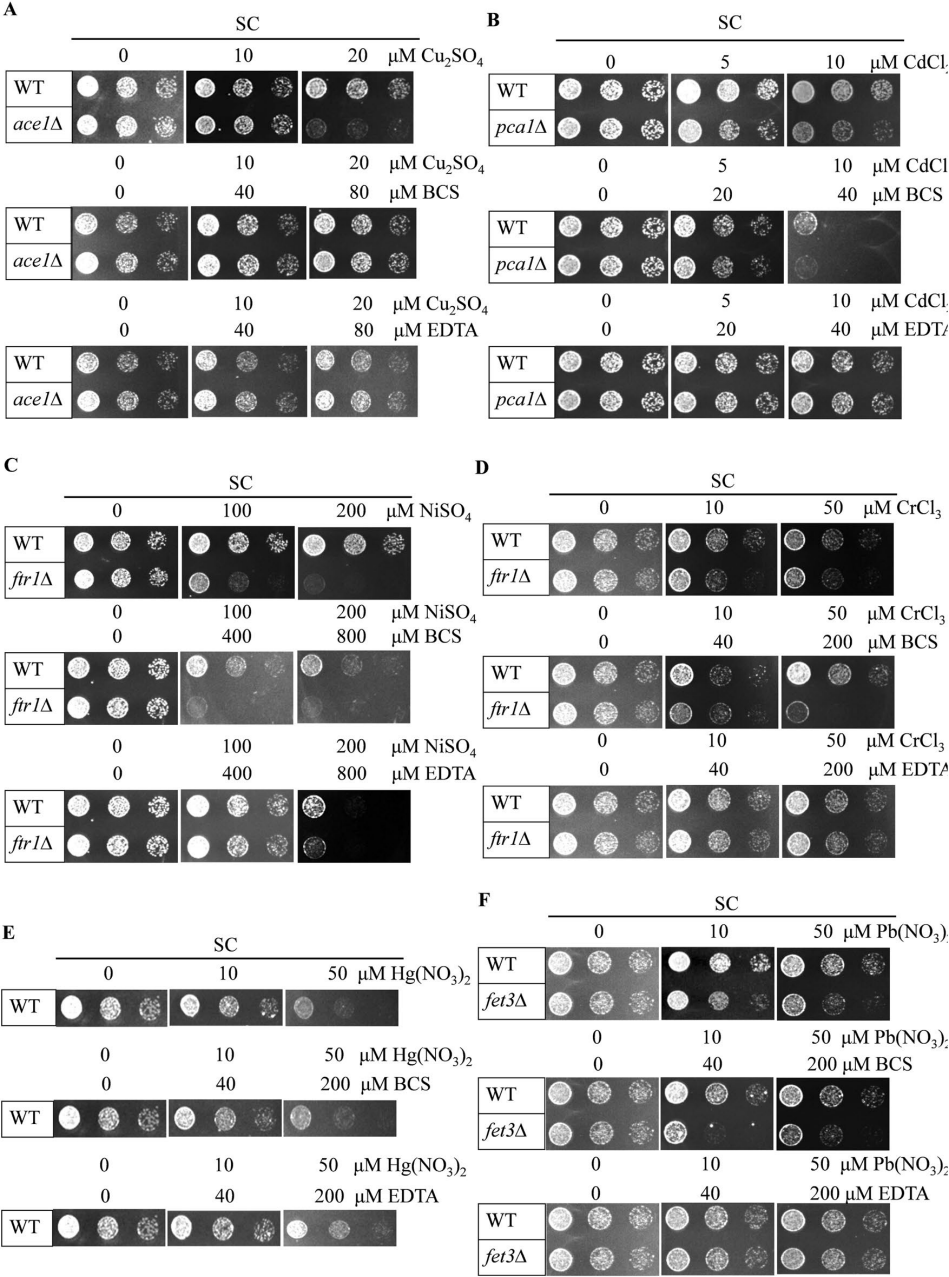
Identification of BCS binding specificity by biological assays. (**A**) Growth of WT control and *ace1Δ* strains in SC media containing glucose with CuSO_4_ or BCS or EDTA supplementation at the indicated concentrations. (**B**) Growth of WT control and *pca1Δ* strains in SC media containing glucose with CdCl_2_ or BCS or EDTA supplementation at the indicated concentrations. Growth of WT control and *ftr1Δ* strains in SC media containing glucose with NiSO_4_ (**C**) or CrCl_3_ (**D**) or BCS or EDTA supplementation at the indicated concentrations. (**E**) Growth of WT strain in SC media containing glucose with Hg(NO_3_)_2_ or BCS or EDTA supplementation at the indicated concentrations. (**F**) Growth of WT control and *fet3Δ* strains in SC media containing glucose with Pb(NO_3_)_2_ or BCS or EDTA supplementation at the indicated concentrations. Exponentially growing cells were spotted on media and assessed after 3 days. All growth assays were conducted with at least four different clones.

Heavy metal ion chelators with high specificity, low toxicity, strong binding ability and color reaction capability are essential to preparing paper test strips of practical use in the detection of trace heavy metal ions. Therefore, screening methods are important to discover new chelators that meet our requirements. In this study, we developed a biological assay based on yeast mutants’ sensitivities to heavy metal ions. To determine the specificity of BCS for copper, we assessed whether metal-induced toxicity of several metal-sensitive yeast knockout strains could be recovered by BCS. A non-specific metal chelator EDTA was used as a positive control. *ace1Δ*, *pca1Δ*, *ftr1Δ*, *fet3Δ*, *ftr1Δ* and wild type cell strains were used to test the sensitivities of Cu^+^, Cd^2+^, Ni^2+^, Pb^2+^, Cr^3+^ and Hg^2+^, respectively. WT and knock out mutants were cultured to mid-log phase. Cells were spotted on SC plates supplemented with various amounts of CuSO_4_, CdCl_2_, NiSO_4_, CrCl_3_, Hg(NO_3_)_2_ and Pb(NO_3_)_2_ with or without BCS or EDTA. As expected, *ace1Δ* cells were highly sensitive to CuSO_4_ at 20 μΜ and this growth defect was rescued by supplementation of BCS or EDTA (Fig. 1A). The growth defects induced by other metals, when treated at toxic concentrations to their respective metal-sensitive yeast strains, were not recovered by BCS, but only by EDTA (Fig. 1B–F). These data indicate that metal chelation by BCS is specific to copper, but not other metal ions. The recovery of cell growth under all metal treatment conditions by positive control EDTA demonstrated that growth defects in this assay were indeed due to metal toxicity.

### Identification of optimal BCS-Cu^+^ reaction conditions

**Figure 2:**
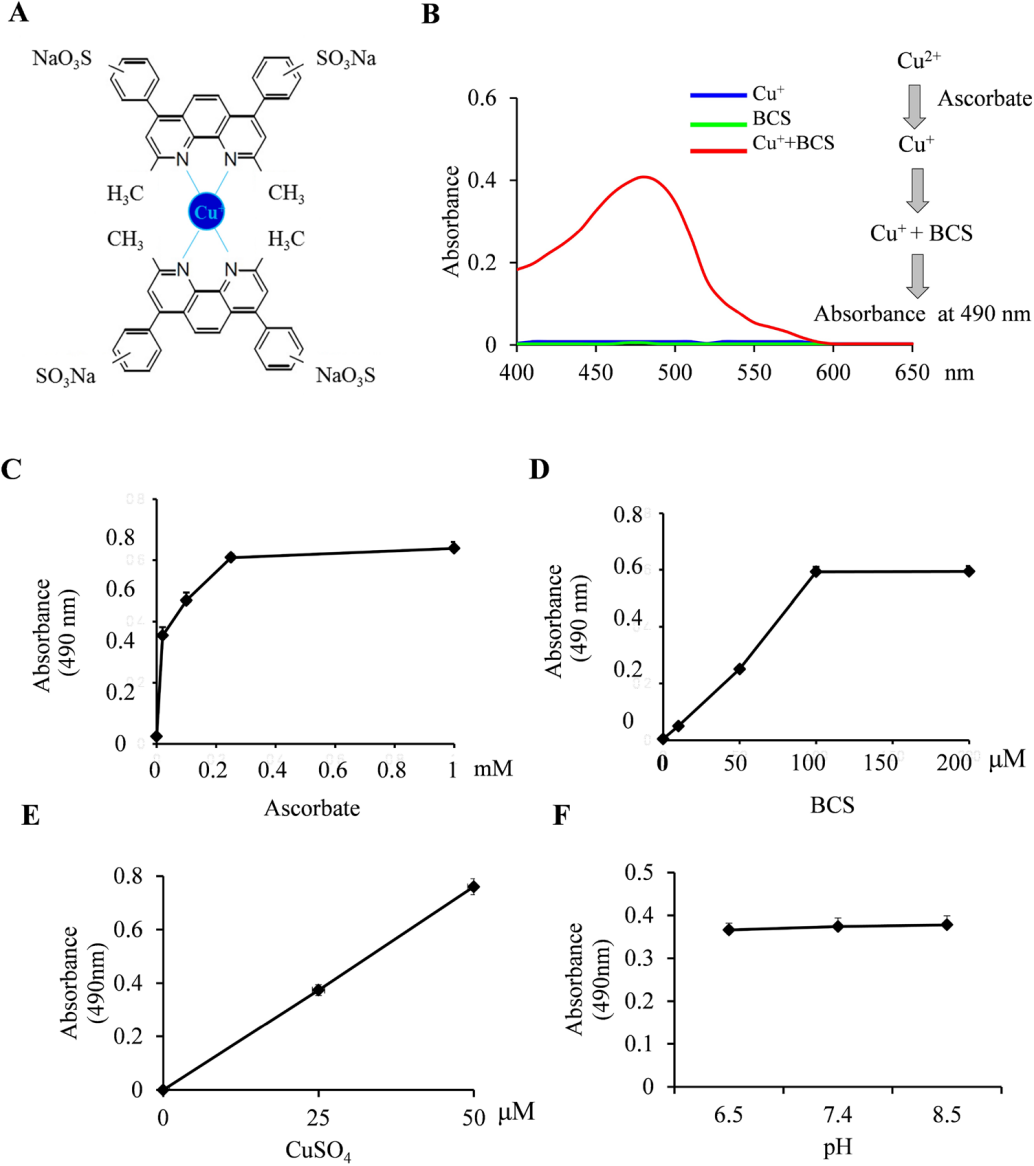
Optimization of BCS-Cu^+^ binding assay. (**A**) Schematic depiction of BCS binding with cuprous ion. (**B**) Absorption spectra of Cu^+^, BCS and Cu^+^-BCS. (**C**) The effect of ascorbate on BCS assay (50 mM Tris–HCl pH 7.4, 200 µM BCS, 25 μM CuSO_4_). (**D**) The effect of BCS on BCS assay (50 mM Tris–HCl pH 7.4, 25 μM CuSO_4_, 1 mM ascorbate). (**E**) The effect of CuSO_4_ on BCS assay (50 mM Tris–HCl pH 7.4, 200 µM BCS, 1 mM ascorbate). (**F**) The effect of pH on BCS assay (200 µM BCS, 25 μM CuSO_4_, 1 mM ascorbate). All assays were conducted at least 6 times. Average ± S.D. is presented. Asterisk * indicates significant difference (* p < 0.05, **p < 0.01).

A previous report showed that BCS binds Cu (I) in a 2:1 ratio (Fig. 2A)^34^. The BCS-Cu^+^ complex was scanned by spectrophotometer and the maximum absorbance was found at 490 nm (Fig. 2B). To optimize the BCS-Cu^+^ reaction conditions, we tested the effects of ascorbate, BCS, CuSO_4_ and pH. The 490 nm absorbance was highest when the ascorbate concentration was increased to 0.25–1 mM under the conditions of 25 μM CuSO_4_ and 200 μM BCS (Fig. 2C). For the BCS effect, 100–200 μM BCS was optimal to bind with 25 μM CuSO_4_ (Fig. 2D). The absorbance was proportional to the concentration of copper in the presence of sufficient BCS and ascorbate (Fig. 2E). There was minimal effect of pH on the absorbance from pH 6.5–8.5 (Fig. 2F). Based on these results, we chose an optimal reaction system for BCS-Cu^+^ binding to consist of 1 mM ascorbate, 200 μM BCS and pH 7.4.

### Chemical identification of BCS binding specificity by spectrophotometer

**Figure 3:**
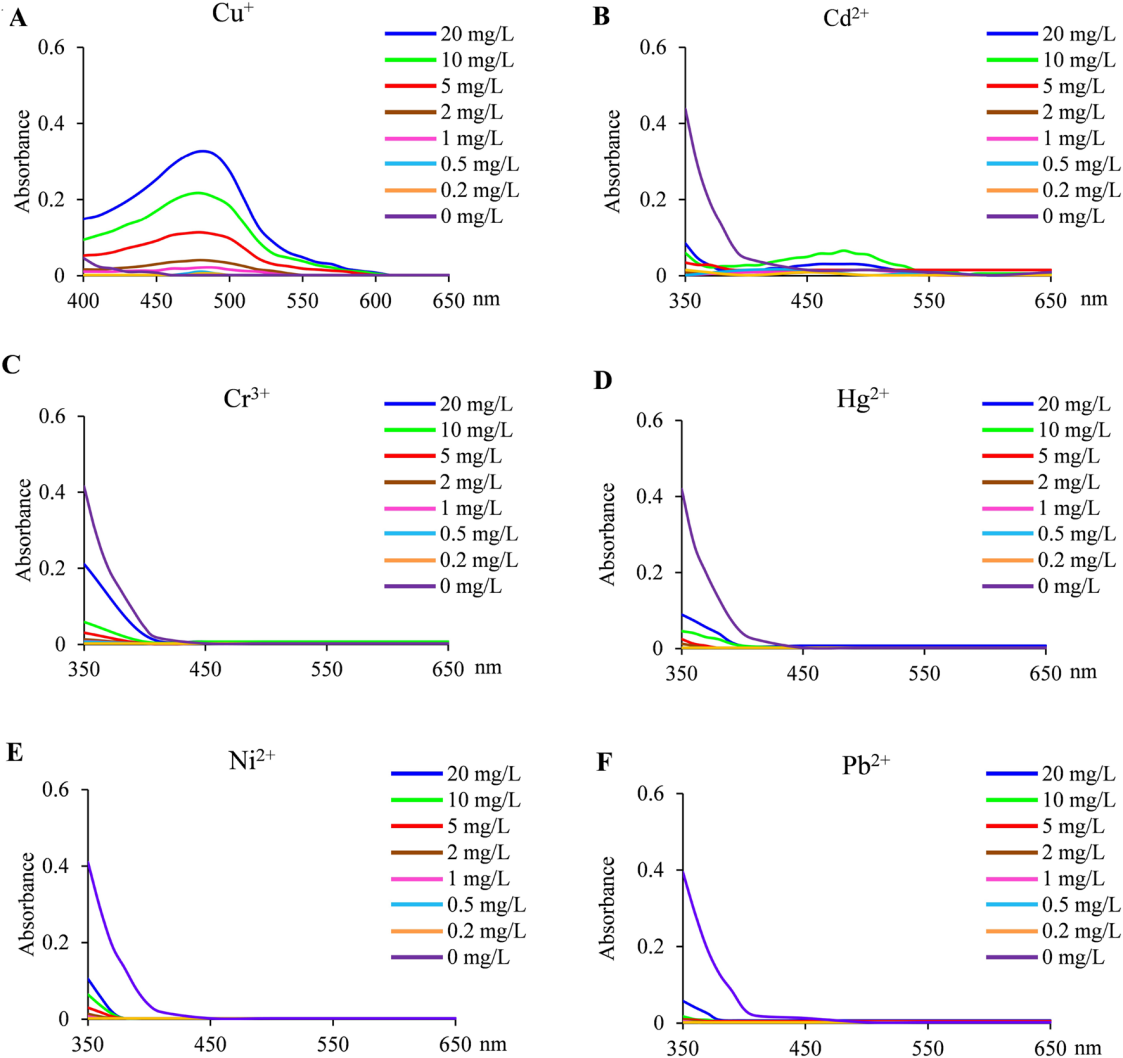
Identification of BCS binding specificity by spectrophotometer. Absorbance spectrum (350–650 nm) measurements under optimized reaction conditions (50 mM Tris–HCl pH 7.4, 200 μM BCS, 1 mM ascorbate) of BCS binding with Cu^+^ (**A**), Cd^2+^ (**B**), Cr^3+^ (**C**), Hg^2+^ (**D**), Ni^2+^ (**E**) and Pb^2+^ (**F**) by spectrophotometer.

We developed a biological assay and demonstrated BCS exhibits a strong specificity to copper. To further confirm this finding and the new biological assay, a chemical identification by spectrophotometer was employed to verify BCS binding specificity. The reaction system was 50 mM Tris–HCl pH 7.4, 200 μM BCS, 1 mM ascorbate and different concentrations of metal ions. The reaction system was mixed with CuSO_4_, CdCl_2_, CrCl_3_, Hg(NO_3_)_2_, NiCl_2_ and Pb(NO_3_)_2_ at concentrations from 0–20 mg/L, and absorbance was monitored. We were able to follow the change in absorbance at 490 nm in line with the introduction of copper (Fig. 3A). The other metal ions, including Cd^2+^ (Fig. 3B), Cr^3+^ (Fig. 3C), Hg^2+^ (Fig. 3D), Ni^2+^ (Fig. 3E) and Pb^2+^ (Fig. 3F) were not able to affect the absorbance at 490 nm. These results were consistent with those of the biological assays. Therefore, chemical identification further confirmed that BCS exhibits a strong specificity to copper and demonstrated the accuracy and validity of biological assay.

### Spectrum method and fabrication of a paper strip for the detection of copper

**Figure 4:**
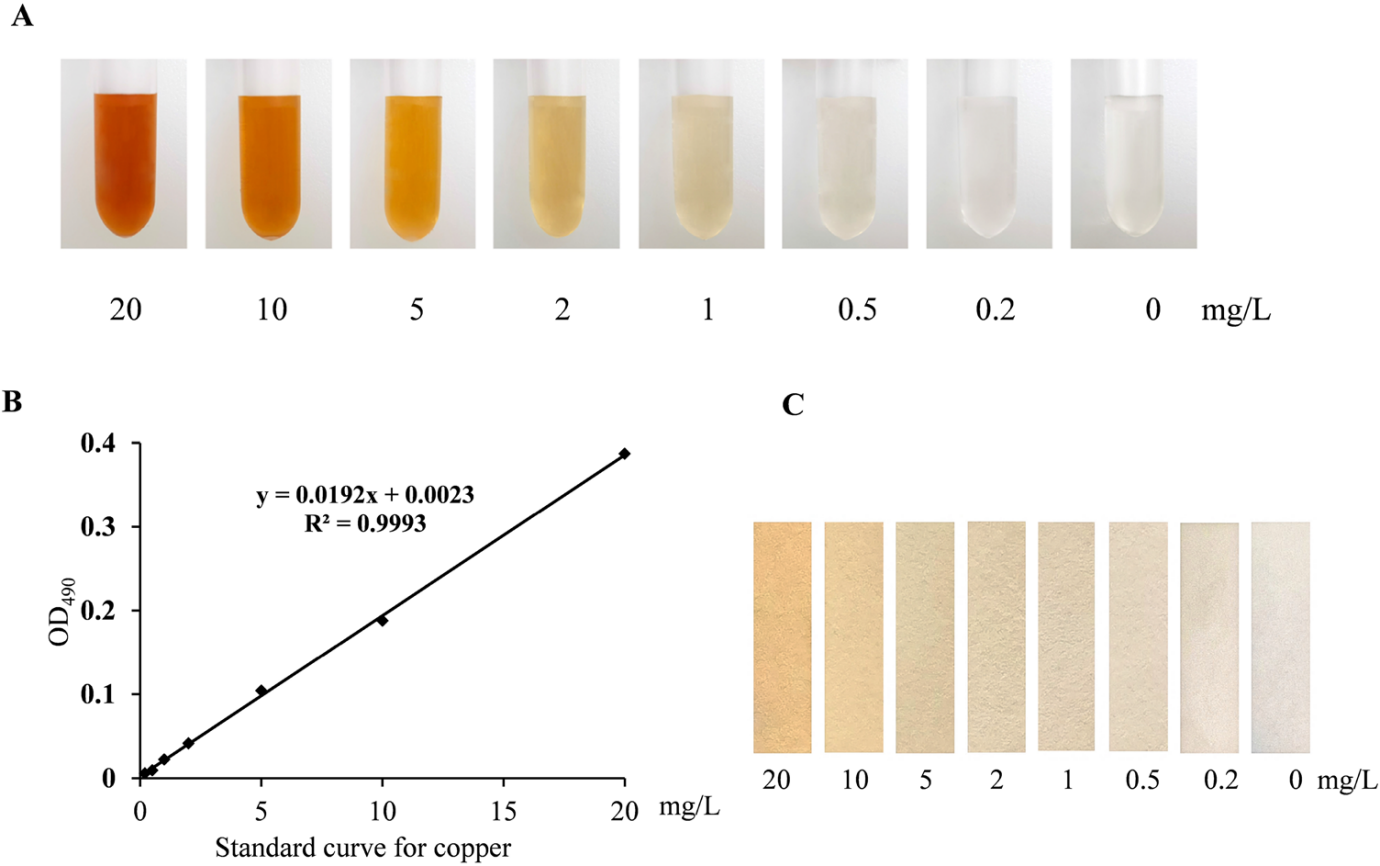
Preparations of standard paper strips and standard curve. Standard solutions of BCS-Cu^+^ were prepared under optimized reaction conditions (50 mM Tris–HCl pH 7.4, 200 µM BCS, 1 mM ascorbate) with varying concentrations of CuSO_4_ (0, 0.2, 0.5, 1, 2, 5, 10, 20 mg/L). (**A**) Standard solutions were imaged in glass test tubes. (**B**) A standard curve of BCS-Cu^+^ was prepared by measuring the absorbance at 490 nm over concentration. (**C**) Paper strips (filter paper, 0.5 × 6 cm) were soaked in standard solutions of BCS-Cu^+^, dried for 1 min and visualized against a white background.

The BCS-Cu^+^ complex generated absorption intensity at 490 nm in proportion to copper content by spectrophotometry^19–21^. In order to accurately obtain the relationship between the reaction chroma and the concentration of copper, the optimal reaction system (50 mM Tris–HCl pH 7.4, 200 μM BCS, 1 mM ascorbate) was mixed with different concentrations of CuSO_4_ from 0–20 mg/L (Fig. 4A). The solution color deepened as the copper concentration increased. The color of solution as low as 0.5 mg/L changed significantly relative to the control color even when judged by naked eyes (Fig. 4A). The solutions were measured by spectrophotometer at 490 nm and a standard curve (y = 0.0192x + 0.0023, R^2^ = 0.9993) of copper and absorbance was established (Fig. 4B). The paper strips (0.5 cm × 6 cm) were soaked into these solutions and dried one minute for color display (Fig. 4C). The color of the test strip gradually deepened as the concentration of copper increased (Fig. 4C). In fact, the color of 0.2 mg/L paper still showed change although it was not very obvious (Fig. 4C). These results demonstrated a novel colorimetric paper strip method with a 0.5 mg/L detection limit (food copper detection limit ≤ 10 mg/kg, GB15199-94) and an optimized spectrum method are successfully established for rapid detection of copper ion.

### Copper measurements of fruits and vegetables by paper strip and optimized spectrum based on BCS-Cu^+^ color reaction

**Figure 5:**
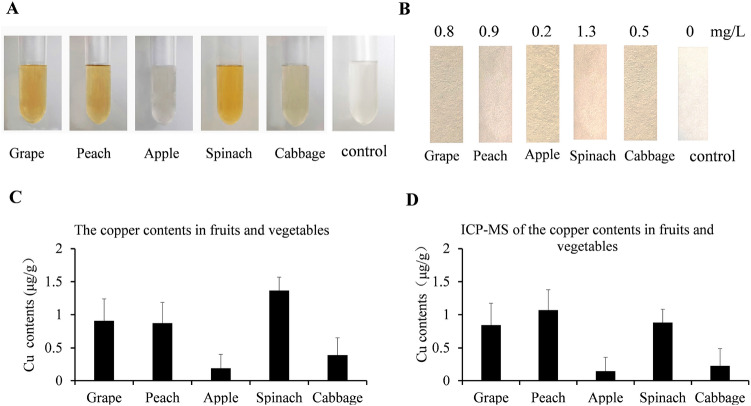
Applications of paper strip and optimized spectrum based on BCS-Cu^+^ color reaction in fruits and vegetables. 0.1 g samples of several common fruits and vegetables (grape, peach, apple, spinach, cabbage) were collected and digested in 10% nitric acid for BCS assays. (**A**) Solutions of the indicated fruits and vegetables were visualized in glass test tubes to demonstrate the color development in solution. Cu contents of indicated fruits and vegetables were determined by paper strips (**B**), optimized spectrum method (**C**) or ICP-MS (**D**).

To test the application of paper strip detection and the optimized spectrum method, grape, peach, apple, spinach and cabbage were purchased from the market. 0.1 g of each sample were smashed and dissolved in 50 μL 10% nitric acid at room temperature for 20 min and subsequently the whole solution was taken into the reaction system for color development, 490 nm measurement and paper test. Compared with the blank control, the five sample solutions displayed obvious color changes (Fig. 5A). The paper strip assays showed that the copper contents of grape, peach, apple, spinach and cabbage were 0.8 mg/L, 0.9 mg/L, 0.2 mg/L, 1.3 mg/L and 0.5 mg/L, respectively (Fig. 5B). The detection results of grape, peach, apple, spinach and cabbage by the optimized spectrum method at 490 nm were 0.91 μg/g, 0.87 μg/g, 0.19 μg/g, 1.37 μg/g and 0.39 μg/g, respectively (Fig. 5C). The ICP-MS measurements showed that the copper contents of grape, peach, apple, spinach and cabbage were 0.84 mg/L, 1.07 mg/L, 0.15 mg/L, 0.88 mg/L and 0.23 mg/L, respectively (Fig. 5D). The results of the optimized spectrum method and paper strip assay correlated well with those determined by ICP-MS. Collectively, our results indicated both the optimized spectrum method and paper strip assay were able to reliably quantitate copper in foods.

### Characteristics and properties of BCS-AgNPs

**Figure 6:**
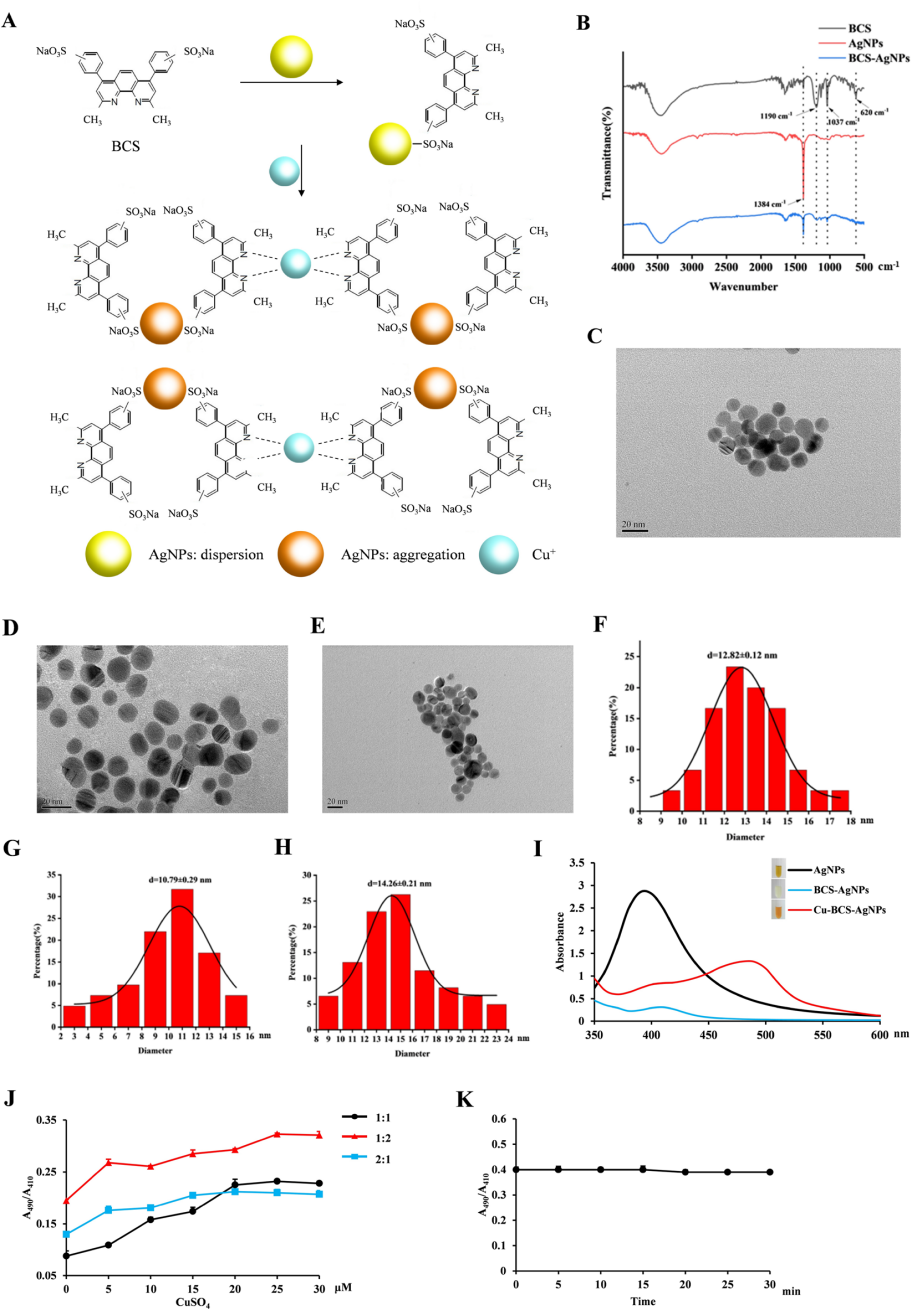
Preparation and characterization of BCS-AgNPs. (**A**) Schematic illustration of Cu^+^ induced colorimetric responses of the BCS-AgNPs (created with Microsoft PowerPoint 2010). (**B**) FTIR spectra of BCS, AgNPs and BCS-AgNPs. TEM images of AgNPs (**C**), BCS-AgNPs (**D**) and Cu-BCS-AgNPs (**E**). Schematic diagram of the particle size distribution of AgNPs (**F**), BCS-AgNPs (**G**) and Cu-BCS-AgNPs (**H**). (**I**) Absorption spectra of AgNPs, BCS-AgNPs and Cu-BCS-AgNPs. (**J**) The effect of the concentration ratio of AgNPs to BCS (1:1, 1:2 and 2:1) on the determination of different concentrations of Cu^+^. (**K**) The effect of time on the determination of 0.5 mg/L Cu^+^ binding to BCS-AgNPs.

Given that nanomaterial is an effective approach to improve detection sensitivity, Ag nanoparticles (AgNPs) was used to increase the color change of standard paper. BCS possesses sulfonic acid groups, which can be easily bound to AgNPs (Fig. 6A). We prepared BCS-functionalized silver nanoparticles and determined characteristics and properties. FTIR spectra of BCS, AgNPs and BCS-AgNPs showed that the characteristic peaks of 620 cm^-1^ and 1190 cm^-1^ belonged to BCS, and the 1384 cm^-1^ belonged to AgNPs (Fig. 6B). The 1384 cm^-1^ peak disappeared after BCS was added. The results indicated that BCS was successfully modified to the surface of AgNPs. TEM images of AgNPs (Fig. 6C), BCS-AgNPs (Fig. 6D) and Cu-BCS-AgNPs (Fig. 6E) revealed the particles were a spheroidal shape. The nano-silver distribution slightly dispersed after the addition of BCS but the aggregation intensified after the addition of copper. The particle size distributions of AgNPs was examined by TEM. Most primary particles were sized within 9–30 nm and the average diameter of particles was ~ 15 nm (Fig. 6F–H). Meanwhile, we optimized Cu-BCS-AgNPs binding conditions and determined the optimal AgNPs:BCS ratio was 2:1 and the optimal reaction time was less than 1 s (Fig. 6I–K). Overall, these results suggested that Cu-BCS-AgNPs are very stable in a nano-particle form and display a special color.

### Preparation of standard paper strips based on Cu-BCS-AgNPs

**Figure 7:**
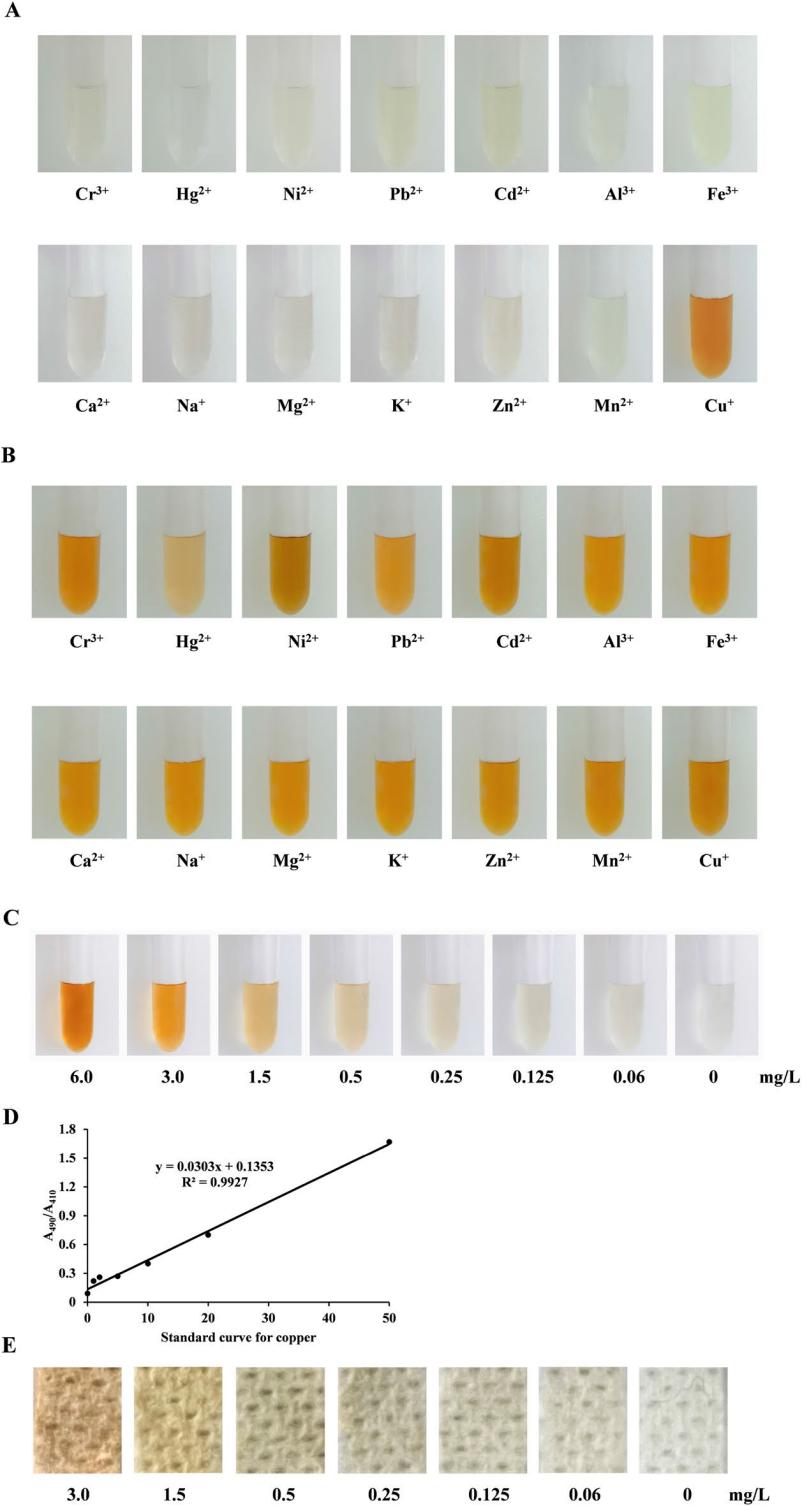
Establishment of standard paper strips based on Cu-BCS-AgNPs. (**A**) Metal specificity assay of BCS-AgNPs. 6.0 mg/L indicated metal ion was added into the BCS-AgNPs system to observe color change. (**B**) Interference assay of other metal ions. 6.0 mg/L indicated metal ion was mixed with Cu^+^ in the BCS-AgNPs system. (**C**) Standard solutions of Cu-BCS-AgNPs were prepared under optimized reaction conditions with indicated concentrations of CuSO_4_. (**D**) The absorbance ratios of Cu-BCS-AgNPs at A_490_/A_410_ were calculated to obtain the standard curve of Cu-BCS-AgNPs. (**E**) Paper strips (filter paper, 0.5 cm × 0.5 cm) were soaked in standard solutions of Cu-BCS-AgNPs, dried for 1 min and visualized against a white background.

In view of Cu-BCS-AgNPs displayed excellent properties of aggregation, color development and stability, we next tested the specificity of Cu-BCS-AgNPs. Thirteen element ions, including Cr^3+^, Hg^2+^, Ni^2+^, Pb^2+^, Cd^2+^, Al^3+^, Fe^3+^, Ca^2+^, Na^+^, Mg^2+^, K^+^, Zn^2+^ and Mn^2+^, were tested using the same BCS-AgNPs system. As shown in Fig. 7A, only Cu^+^, but not other metal ions, resulted in a significant color change (Fig. 7A). In order to evaluate the interference of other metal ions on copper, the 13 metal ions were mixed with the same concentration Cu^+^ and added into the BCS-AgNPs reaction system. The color changes indicated that other metal ions displayed very weak influence on Cu^+^ binding to BCS-AgNPs (Fig. 7B). In order to accurately obtain the relationship between the reaction chroma and the Cu- BCS-AgNPs, the optimal reaction system was mixed with different concentrations of CuSO_4_ from 0–6 mg/L (Fig. 7C). The solution color deepened as the copper concentration increased. The solution color of 0.06 mg/L changed significantly relative to the control color even when judged by naked eyes (Fig. 7C). The solutions were measured by spectrophotometer at 490 nm/410 nm and a standard curve (y = 0.0303x + 0.1353, R^2^ = 0.9927) of copper and absorbance ratio was established (Fig. 7D). The paper strips (0.5 cm × 0.5 cm) were soaked into these solutions and dried one minute for color display (Fig. 7E). The color of the test strip gradually deepened as the concentration of copper increased (Fig. 7E). Consistent with color changes of solutions, the color of 0.06 mg/L paper still showed an obvious change by eye (Fig. 7E). These results demonstrated we have successful developed a sensitive colorimetric paper strip method based on Cu- BCS-AgNPs with a 0.06 mg/L detection limit.

## Discussion

As an essential element of organisms and a widely used industrial raw material, copper is extensively distributed in water, soil and agricultural products^4,35,36^. Therefore, it is necessary to develop rapid and effective methods for the detection of copper contents in the environment and food to ensure environmental safety and human health.

Among all the rapid detection methods for copper ions, paper-based analytical devices (PADs) are becoming a popular research field owing to their portability, on-site detection, simple operation, low cost and multifunctional analysis^10–12^. The mechanisms of these paper analytical test strips mainly utilize the combinations of heavy metal ions and chelators to produce color reactions. Hence, chelators possessing high specificity, low toxicity, strong binding ability and ability to produce a color reaction are crucial to preparing paper test strips. BCS has been employed as a chelator in many fields, such as evaluation of the copper (II) reduction assay^19^, determination of D-glucose and D-galactose levels^37^, development of multi-walled carbon nanotube Cu sensors^18^, and quantitation of Cu and Cu-protein complexes^17,18,21^. During our study of copper ion metabolism, we identified the BCS-Cu^+^ complex showed different intensities of yellow on paper strip and in solution proportional to copper levels. Based on this discovery, we developed a rapid, on-site, low-cost and safe single paper strip detection method for copper and this novel method was applied to detect copper levels of fruits and vegetables.

Chelator specificity is key to the practical application of paper strip detection. BCS has been used as a copper chelator for a long time, but there was no intuitive way to reflect its specificity^17^. In this study, we took advantage of heavy metal sensitive yeast mutants to identify the binding capacity of BCS to various heavy metals. This method intuitively showed that BCS displayed strong binding capacity to copper ions but not other heavy metal ions.

To thoroughly compare our method with some other reported methods, we analyzed their features in terms of sensitivity, specificity, detection time, instrument aids, detection object and cost (Table 1). Overall, our method displayed high sensitivity, easy portability, on-site detection, simple operation, low cost and practical applicability.

**Table 1:** Comparison of rapid detection methods for copper ion.

Method	Sensitivity	Specificity	Detection time	Instrument aids	Detection object	Cost	Reference
This method	0.06 mg/L	High	1 min	No	Fruits, vegetables, water	Lower	
Colorimetric	0.17 mg/L	Good	1 min	Yes	Waste water	Low	^11^
Colorimetric	6.4 mg/L	Good	A few minutes	No	Aqueous solutions	Low	^44^
Colorimetric	0.64 mg/L	Good	A few seconds	No	Aqueous solutions	Lower	^45^
Spectrophotometer	0.64 mg/L	High	A few minutes	Yes	Aqueous solutions	Low	^46^
Spectrophotometer	4.13 μg/L	High	A few minutes	Yes	Aqueous solutions	Low	^47^
Fluorescence	2.98 μg/L	High	8 s	Yes	Wine	Low	^48^
Nanomaterial	0.96 mg/L	Medium	20 min	No	Aqueous solutions	Low	^49^
Nanomaterial	3.2 mg/L	Medium	24 h	No	Aqueous solutions	Lower	^50^
Nanomaterial	76.8 μg/L	High	10 min	Yes	Aqueous solutions	Low	^51^
Electrochemistry	6.4 μg/L	Medium	150 s	Yes	Aqueous solutions	Low	^52^

Although the detection results of grape, peach, apple, spinach and cabbage by the optimized spectrum method and paper strip assays correlated well with those determined by ICP-MS, there are still some aspects worth improving. The first is the sample pretreatment. Concentrated acid digestion is the most common pretreatment for food materials and concentrated HNO_3_ is preferentially used owing to its high purity and broad scope of oxidation ability^38^. Despite the high digestion efficiency of concentrated HNO_3_, a high residual acidity and the production of highly acidic digests do not comply with green analytical chemistry requirements^39,40^. To address these shortcomings, 10% diluted HNO_3_ was employed to digest in smashed samples for copper analysis. These results indicated that this is a feasible method to completely digest samples of fruits and vegetables, such as grape, peach, apple, spinach and cabbage. But if we desire to expand the application scope of these novel paper strip and optimized spectrum methods to less digestible samples, such as rice, a more effective strategy must be developed. Recently, a microwave-assisted digestion method using diluted HNO_3_ was developed for determination of heavy metals in rice^39^. Lee et al. reported that a diluted nitric acid and hydrogen peroxide mixture is another way to optimize acidic digestion while minimizing environmental impact^40^.

The second aspect to further improve is the detection limit, specifically, to enhance the color intensity under low copper levels. Nanomaterials with unique chemical and electrochemical properties show extensive applications in increasing detection sensitivity^41,42^. Borah et al. reported that GA-AuNP@Tollens’ complex as a highly sensitive plasmonic nanosensor strengthened the detection of formaldehyde and benzaldehyde in preserved food products^43^. The use of novel nanomaterials with high signal strength is considered as the most effective strategy to improve the detection limit of the BCS-Cu^+^ based paper strip test but these nanomaterials may increase environmental impact and cost. In this study, Ag nanoparticles (AgNPs) was used to improve the color change of standard paper and increase detection sensitivity. We developed a sensitive colorimetric paper strip method based on Cu- BCS-AgNPs with a 0.06 mg/L visual detection limit.

The third aspect worth improving is the chromatography paper quality. In this research, even the use of common filter paper to display the color change still yielded reasonable detection. Different filter papers may be tried to enhance color quality of the BCS-Cu^+^ complex.

To avoid subjectivity in color judgment, a smartphone can be used as a detector for color judgment albeit with the increase in detection cost^11^. However, if subsequent studies improve the quality of the test paper, it may be possible to accurately judge the color change with the naked eye, similar to the pH paper test strip, and therefore it would not be necessary to use additional equipment to assist color judgment.

In conclusion, the BCS-Cu^+^ complex displayed different intensities of yellow proportional to copper levels in our reaction solution and on paper strip, and this reaction was harnessed to develop a rapid, on-site, low-cost and safe single paper strip test for copper detection which was successfully applied to determine copper contents of fruits and vegetables.

## Data Availability

The data underlying this article are available from the corresponding author upon request.
